# Impact of the Main Cardiovascular Risk Factors on Plasma Extracellular Vesicles and Their Influence on the Heart’s Vulnerability to Ischemia-Reperfusion Injury

**DOI:** 10.3390/cells10123331

**Published:** 2021-11-27

**Authors:** Miłosz Majka, Marcin Kleibert, Małgorzata Wojciechowska

**Affiliations:** 1 Laboratory of Centre for Preclinical Research, Department of Experimental and Clinical Physiology, Medical University of Warsaw, Banacha 1b, 02-097 Warsaw, Poland; miloszmajka98@gmail.com (M.M.); marcin.kleibert@gmail.com (M.K.); 2 Invasive Cardiology Unit, Independent Public Specialist Western Hospital John Paul II, Daleka 11, 05-825 Grodzisk Mazowiecki, Poland

**Keywords:** extracellular vesicles, exosomes, microvesicles, ischemia-reperfusion injury, myocardial infarction, diabetes, obesity, smoking, physical activity, miRNA

## Abstract

The majority of cardiovascular deaths are associated with acute coronary syndrome, especially ST-elevation myocardial infarction. Therapeutic reperfusion alone can contribute up to 40 percent of total infarct size following coronary artery occlusion, which is called ischemia-reperfusion injury (IRI). Its size depends on many factors, including the main risk factors of cardiovascular mortality, such as age, sex, systolic blood pressure, smoking, and total cholesterol level as well as obesity, diabetes, and physical effort. Extracellular vesicles (EVs) are membrane-coated particles released by every type of cell, which can carry content that affects the functioning of other tissues. Their role is essential in the communication between healthy and dysfunctional cells. In this article, data on the variability of the content of EVs in patients with the most prevalent cardiovascular risk factors is presented, and their influence on IRI is discussed.

## 1. Introduction

**Figure 1:**
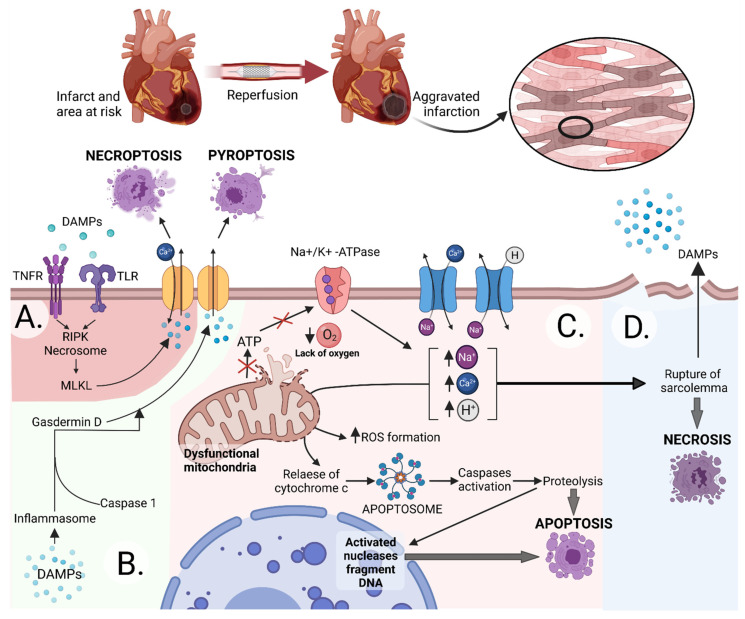
Molecular mechanisms involved in ischemia-reperfusion injury. All cell death types are involved in IRI, including regulated such as necroptosis (**A**), pyroptosis (**B**), apoptosis (**C**), and unregulated such as necrosis (**D**). (**A**) The activation of TNF-α (Tumor Necrosis Factor-alpha) receptors and TLRs (Toll-like Receptors) by DAMPs (Damage-associated Molecular Patterns), which are released during mitochondria and cell rapture, promote necroptosis. Stimulation of Tumor Necrosis Factor receptor recruits RIPKs (receptor-interacting serine/threonine-protein kinases) by proteins associated with the receptor. The RIPKs form the necrosome which phosphorylate the MLKL (mixed-lineage kinase domain-like proteins). Phosphorylated MLKL activates the pores which permeabilize plasma [15]. (**B**) DAMPs stimulate inflammasome formation, which activates caspases, leading to the formation of gasdermin-dependent pores in the membrane. (**C**) The lack of oxygen induces failure of ion pumps, acidosis, and Ca^2+^ overload [16]. In anaerobic glycolysis, the production of H^+^ is increased. The 2Na^+^/Ca^2+^ and the Na^+^/H^+^ ion exchangers remove sodium excess and increase calcium and hydrogen levels [17]. The elevated Ca^2+^ load induces reactive oxygen species (ROS) production and activates phospholipases and proteolytic enzymes which stimulate the release of cytochrome c from the mitochondria and apoptosis in consequence. In addition, elevated Ca^2+^ and inorganic phosphate levels stimulate the opening of the mitochondrial permeability transition pore (mPTP), which in turn causes mitochondrial matrix swelling and outer membrane rupture [18,19]. (**D**) Elevated ion levels and swelling of the cell promote rupture of the cell membrane and necrosis. These pathways and many others not mentioned due to this article’s limitations are responsible for IRI. Heusch recently published a more extensive description of the IRI mechanism [12]. Reperfusion injury can be regulated at any stage by many molecules, such as miRNA (micro ribonucleic acid/miR) and proteins. Created with BioRender.com.

Cardiovascular diseases are a major cause of death worldwide; risk estimation and interventions to reduce mortality are essential during management with patients [1]. Initiated in 1984, the Framingham Heart Study assessed the main risk factors of cardiovascular mortality presented in the SCORE charts (Systematic COronary Risk Evaluation) estimating the 10-year risk of death. It takes into account age, sex, systolic blood pressure, smoking, and total cholesterol level [2,3,4]. In addition, increasing average weight, low physical activity, concentration of low-density lipoprotein (LDL), and incidence of diabetes mellitus were identified as major modifiable risk factors in the further analysis of this study [5,6,7]. The majority of cardiovascular deaths are associated with acute coronary syndrome (ACS), especially ST-elevation myocardial infarction (STEMI) [8]. Due to improvements in diagnosis and treatment, the death rate has decreased in the last decades. Unfortunately, despite a further decline in the door-to-balloon time (the time between arrival at an emergency department and reperfusion), the one-year death rate associated with STEMI has not fallen and is on the level of around 4–6% in the latest clinical trials and analysis [9]. Early reperfusion saves the ischemic myocardium from infarction. However, it also induces a specific additional component of irreversible injury, namely ischemia-reperfusion injury (IRI), and this alone can contribute up to 40% of total infarct size following coronary artery occlusion [10]. The mechanism of IRI is not fully understood. Some risk factors make the myocardium more or less sensitive to this damage. Many pathways and molecules are involved, but a few play a crucial role (Figure 1). Further decline in mortality related to myocardial infarction (MI) depends on appropriate interventions preventing or limiting reperfusion injury [11]. One of these is ischemic conditioning (IC)–induction of short ischemic and reperfusion periods of the myocardium before the onset of MI or during reperfusion. A related method is remote ischemic conditioning (RIC), which consists of applying cycles at a remote site [12]. It is not fully understood, but there are an increasing number of studies showing that extracellular vesicles (EVs) can be a crucial player in this process. As EVs can carry content that affects the functioning of recipient cells, their role is essential in the communication between healthy and dysfunctional cells [13]. EVs are present in abundance in the circulation and play an important role in cardiovascular physiology, which they affect in a pleiotropic manner [13,14]. Despite the great interest in these particles, there is not enough data on their role in the pathophysiology and progression of cardiovascular diseases.

In this article, data on the variability of the content of EVs in patients with the most prevalent cardiovascular risk factors is presented. Moreover, the influence of EVs on reperfusion injury is discussed and their potential role in IRI is assessed.

## 2. Extracellular Vesicles

### 2.1. Nomenclature and Biogenesis

**Figure 2:**
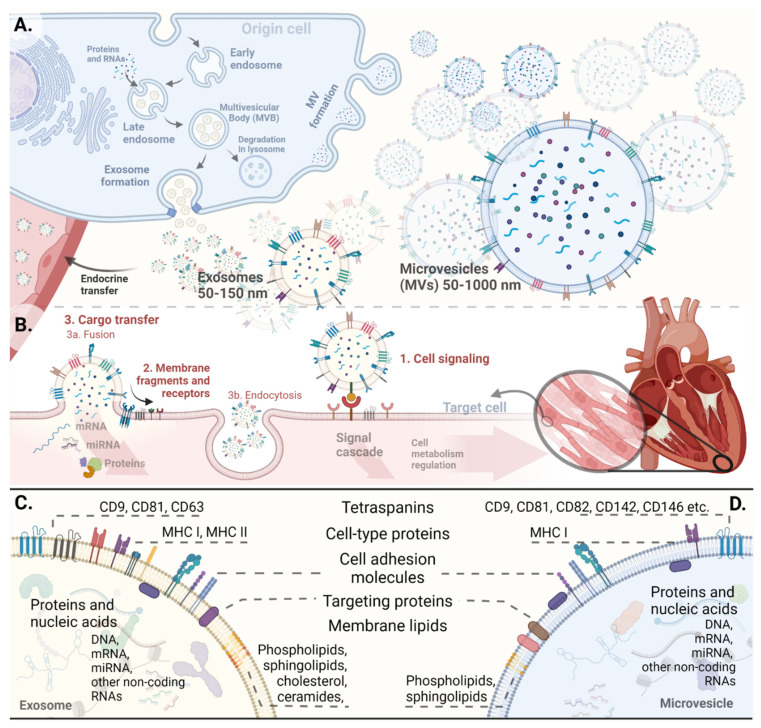
(**A**) Biogenesis of extracellular vesicles (EVs) and their interactions with recipient cells (**B**). (**C**,**D**) The main components of EV membrane and EV cargo; CD—Clusters of Differentiation, MHC—Major Histocompatibility Complex. Created with BioRender.

Extracellular vesicles are a very diverse group due to differences in their origin, release, content, and function, and they can be divided into three main subtypes: exosomes, microvesicles (MVs), and apoptotic bodies. Exosomes and MVs are particularly important in the context of interorgan communication, and they are referred to as EVs in this review (Figure 2). Their size varies within a large range, but it can be assumed that exosomes are vesicles ranging from 30 nm to 150 nm, while microvesicles range from 50 nm to 1 μm [20]. EVs can be released by all cell types. Exosomes are formed by the multivesicular bodies (MVBs) budding into the endosomal lumen, which then fuse with the cell membrane releasing EVs to the extracellular space [21,22,23]. This process in general is regulated by the endosomal sorting complexes required for transport (ESCRT). The formation of microvesicles relies on the outward budding of the cell membrane (Figure 2A). This complex process depends on rearrangements in the cell membrane, in proteins, in the cytoskeleton, and more, which has been discussed by others [24]. The formation route may vary from cell to cell and can follow the above pathways in different ways. These changes in biogenesis associated with various origin translate into different contents and function of individual EVs [24].

### 2.2. EV Characterization

EVs contain many proteins, enzymes, lipids, and nucleic acids. Their biologically active material is stored inside them or is a part of their lipid bilayer membrane, functioning as receptors or ligands [24]. Some molecules are specific for the vesicles and can be used as markers, while other molecules can be typical for the origin cell (Figure 2C,D). EVs contain a variety of lipids with bioactive or structural and trafficking functions. Their composition may change the properties of EVs, which take part in disease processes [25]. Different contents of cholesterol, sphingomyelin, phosphatidylserine, or unsaturated fatty acids in the membrane are associated with different degrees of stiffness, and thus–resistance in circulation, and the possibility of fusion with cell membranes [26]. The most intriguing content of EVs are the RNAs, especially mRNA and miRNA [27,28,29,30,31]. MicroRNAs are short non-coding RNAs of about 22 nucleotides that mediate gene silencing by guiding Argonaute proteins to target sites in the 3′ untranslated region of the mRNAs. Nucleic acids are the major source of variation of the EV, and the metabolic cell’s status affects both their profile and quantity [32]. Moreover, cells selectively package these molecules under various conditions, but the mechanism is still poorly understood. Recent studies showed that it may depend on the sequence of nucleic acids [33]. Both mRNA, which can be translated into proteins in the target cells, and miRNA keep their functions in the recipient cells. Inflammation, hypoxia, or hyperglycemia impact the miRNA profile inside EVs and may lead to changes in metabolism by activation or suppression of multiple molecular pathways [33,34]. Recent studies showed that the differences in EV status among patients may play a role in the pathogenesis of many cardiovascular diseases and can be a significant factor impacting a patient’s prognosis and outcome [35,36,37,38,39]. 

### 2.3. Uptake and Function

The interaction of EVs with cells occurs in several ways: (1) by affecting the cell surface, its receptors and ligands, (2) by implanting membrane fragments, or (3) by delivering the EV cargo to recipient cells by fusion or endocytosis [21,34] (Figure 2B). The internalization of EVs into other cells is still poorly understood, but it probably depends on factors such as the acidity of the microenvironment and the oxygen concentration [26,40,41]. The mechanism by which EVs reach specific cells needs further investigation.

The understanding of the biological function of EVs is still evolving. According to the original hypothesis, EV production was a method for cells to dispose of unnecessary material. Subsequent studies considered EVs mainly in terms of the immune system, cancers, and neurodegenerative diseases [42,43,44]. Over time, EVs became known as the new cell-to-cell communication mechanism, with superiority over other forms due to their ability to transfer bioactive cargo and horizontal gene transfer [14,20,45]. Their function may be various depending on the cellular origin. For example, EVs released by immune cells take part in antigen presentation or modulation of the immune response, while platelet-derived EVs are important factors in angiogenesis and coagulation [41]. EVs have been also described as important factors in cardiac cell-to-cell communication. Their role includes cardiac protection as well as mediating pathological changes including ischemia-reperfusion injury [46]. 

### 2.4. Vs and MI

In the case of myocardial infarction, the potential function of EVs has been best studied in the field of regenerative medicine [13,47]. It has been known for a decade that mesenchymal stem cells (MSCs) have great regenerative capabilities. EVs are the important factor responsible for this phenomenon, particularly by delivering microRNA [47,48,49,50,51,52,53,54]. Indeed, stem cell derived EVs have many promising therapeutic effects in the field of chronic coronary syndrome by attenuating reperfusion injury and remodeling as well as increasing cardiomyocyte regeneration and angiogenesis [55]. 

Moreover, cardiomyocytes and other cells of the heart release different types of EVs under various conditions, including ischemia. They are considered as biomarkers and are of great importance in the pathophysiology of the heart, such as post-MI fibrosis and hypertrophy [32,56,57,58]. 

Vicencio et al. showed that endogenous plasma exosomes expressing heat shock protein (HSP) 70 have cardioprotective effects against ischemia-reperfusion injury by the activation of the pro-survival signaling pathway involving toll-like receptor (TLR) 4 and various kinases including extracellular signal-regulated kinases, leading to the activation of HSP27 [59]. Circulating exosomes were also previously proposed as a key mediator in transferring the protection effect during RIC [60]. The mechanism of RIC is still not fully understood. Currently, neuronal and humoral signal transfer can be recognized, for which EVs and miRNA are thought to be important. The RIC procedure increases the concentration of circulating exosomes, which attenuate ischemia-reperfusion injury in rat models by transferring miRNA or activating cardioprotective pathways [59,61,62,63]. However, data from randomized controlled trials assessing the number and content of EVs after RIC in humans is inconsistent [64,65,66].

Recent studies show that the protective effect of EVs on myocardial IRI can be reduced or even inverted in the setting of disease [67,68,69]. Indeed, EVs have both harmful and beneficial effects, and some pathologies may contribute to the loss or gain of the functions of EVs [70]. Their relationship with other diseases and with well-known cardiovascular risk factors is still still not understood.

## 3. Cardiovascular Risk Factors

### 3.1. Diabetes Mellitus and Obesity

It is known that hyperglycemia or a previous diagnosis of diabetes mellitus (DM) increases susceptibility to IRI [71,72,73]. Patients with DM experience worse clinical outcomes, which partly result from more severe damage associated with reperfusion [74]. This effect may be directly related to hyperglycemia or changes in insulin levels, which has an impact on the number and content of EVs. Due to the different pathogenesis of both types of DM, it is worth discussing their effect on IRI separately. Moreover, obesity is associated with insulin resistance, and a high-fat diet animal model is commonly used to investigate the pathogenesis not only of obesity but also of type 2 diabetes. Therefore, the results of most studies concern the their collective influence.

#### 3.1.1. DM Type 1

This type of DM results from the decreased synthesis of insulin due to the autoimmune damage of pancreatic β-cells. Basic research has shown that type 1 DM (T1DM) could enhance the IRI and attenuate the action of protective mechanisms [75,76]. Streptozotocin-induced diabetic mice had a significantly higher death rate post IRI than wild-type mice (death rate 69% vs. 9%; *p* < 0.05), and treatment with insulin reduced the damage size [77]. The growing body of data suggests that EVs can play a role in DM pathogenesis as an immune modulator [78,79]. The altered number and cargo of EVs in serum and urine are associated with the progression and development of complications of DM. Indeed, a higher level of HbA1c (glycated haemoglobin) is related to greater differences in EV content [80,81,82]. Studies report that serum obtained from patients with T1DM had a significantly higher number of platelet-derived, monocyte-derived, and endothelium-derived MVs than the serum from healthy volunteers [83,84]. The impact of these differences on IRI is still not understood, however studies show that MVs may play a role in remote conditioning by transporting cardioprotective signals [85]. In addition, an increase in microparticle shedding can be reduced by 10 days of aspirin therapy [86]. 

Expression of miRNAs inside EVs is dysregulated in T1DM patients [87,88]. Dysregulation of 316 out of 1008 total miRNAs was also observed in diabetic hearts [89]. Some of them can modify the IRI [90,91], but a further assessment of the significance of this is needed as most studies have only been conducted on streptozotocin-induced diabetic models. Furthermore, EVs loaded with miR-21 were assessed as an early biomarker of DM type 1 in children, as their number increases with the duration of the disease and this induced apoptosis of the Langerhans islets [92]. Interestingly, the same miRNA has a protective function against IRI and has been suggested as a potential pharmacological target in IRI in patients with DM type 2 [91]. On the other hand, in vitro studies showed that Langerhans islets incubated with cytokines (the model of T1DM) could release EVs loaded with miR-29b, which can promote apoptosis and the inflammatory process in reperfusion injury [93]. Moreover, EVs obtained from a similar culture can activate the T helper lymphocytes, which take part in the pathogenesis of IRI, especially in late complications such as fibrosis [94,95,96]. 

#### 3.1.2. DM Type 2 and Obesity

Obesity has become an alarming worldwide health problem. Because obesity causes a crucial dysfunction of the adipose tissue metabolism, it contributes to system-wide pathologies, including type 2 diabetes and cardiovascular problems. Overweight and obesity are important factors contributing to all-cause mortality. Their association with mortality following acute myocardial infarction seems to be inverse, which is known as the obesity paradox [97]. However, overweight, obesity, and type 2 diabetes themselves increase the cardiometabolic risk. Diet or weight management is an essential component of secondary prevention. Moreover, cardiovascular risk further increases when both obesity and diabetes coexist.

Obesity affects the functioning of the body in many ways, one of which is the endocrine and paracrine function of adipose tissue [98]. It involves many bioactive mediators, such as hormones, adipokines, and inflammatory factors. They modify the functioning of the heart and its response to ischemia and reperfusion. Some hormones, such as adiponectin, leptin, TNF-α, plasminogen activator inhibitor-1, and visfatin, are associated with a higher cardiovascular risk and insulin resistance. The distribution of tissue is also an important factor in DM development, as visceral fat secretes more substances than subcutaneous fat, which can modify carbohydrate metabolism and promote insulin resistance [99,100]. In addition, chronic inflammation plays a causal role [101]. The secretion of multiple factors by hypertrophic adipocytes, such as resistin, IL-6, IL-8, and TNF-α leads to the phosphorylation of insulin-related receptors, causing the alteration of its signaling [100].

Adipocyte-derived EVs can act as a mode of communication between adipose tissues and other cells, impairing their function in obesity [102,103]. Adipose tissue is also an important source of exosomal miRNA, regulating metabolism in distant tissues, and considered to be a new form of adipokine [104]. The composition and number of EVs from adipose tissue, including their level in the blood and the profile of their miRNA, differ in obesity and diabetes [105,106,107,108]. The number of EVs is usually increased in patients with type 2 DM, even when it is analysed independently of obesity status. It also correlates with hypertension, arterial stiffness, diabetic kidney disease, and macro-/micro-angiopathy [109,110,111,112,113,114,115,116]. In addition, bariatric surgery and glycaemic control restoration alter the number and cargo of EVs [117,118].

Considering these changes, EVs have become an important element in understanding the mechanism linking obesity and type 2 diabetes with other diseases and complications in different organs [119,120,121,122,123,124,125]. Studies showed that circulating exosomal miRNA, altered by obesity and diabetes, can worsen heart function, and their inhibition has a therapeutic effect [126,127,128]. The cardioprotective effect of plasma exosomes by the HSP70/TLR4 pro-survival axis is dramatically altered in a state of hyperglycaemia as shown by Davidson et al. (2018). These and other pro-survival pathways are diminished in cardiomyocytes from diabetic rats, which would inhibit the proliferation and angiogenesis of cardiomyocytes and promote apoptosis [59,67,129,130,131]. As EVs play a protective role by mediating RIP signals, this study may also explain the failure of protection in the heart by RIP among diabetic animals [69].

As was recently shown by Gan et al. (2020), EVs mediate the detrimental effect of dysfunctional adipose tissue by exacerbating susceptibility of the myocardium to IRI [68]. Exosomes derived from the serum and adipose tissue of high-fat diet mice increase cardiomyocyte death due to ischemia-reperfusion injury. It is associated with the inhibition of antiapoptotic and cardioprotective factors, mainly 5′AMP-activated protein kinase alpha, by miR-130b-3p. The amount of this microRNA is significantly increased in the EVs, the adipose tissue, and the heart of diabetic animals. Moreover, IRI can be attenuated by the administration of the EV generation inhibitor or miR-130b-3p inhibitor, therefore targeting EV-mediated communication between the adipose tissue and the heart may be a novel strategy attenuating the diabetic exacerbation of myocardial infarction size. In contrast, EVs from lean animals had the opposite, beneficial effect by the attenuation of IRI.

There are many other dysregulated circulating miRNAs in obesity and diabetes, and their expression differs between obese patients with and without type 2 diabetes [132]. Some of them were reported to play an essential role in obesity-related heart diseases and cardiovascular complications [87,133]. A list of miRNAs, which have been reported to affect the IRI, are presented in the table below (Table 1 and Table 2). Wang et al. showed that EVs derived from the cardiomyocytes of obese diabetic rats play a role in spreading disease to neighbouring cells and inhibit angiogenesis by transferring higher levels of miR-320 and lower levels of miR-126 than healthy controls [134]. Similar changes in microRNA are observed in EVs derived from the adipose tissue and plasma of obese patients [37,132,135,136]. The overexpression of miR-320 can induce cardiomyocyte apoptosis by targeting A kinase interacting protein 1, HSP20, and insulin-like growth factor-1, whereas its suppression alleviates IRI [137,138,139]. The opposite properties are shown by miR-126, which can attenuate myocardial susceptibility to IRI, and miR-126 downregulation during reperfusion aggravates cardiomyocyte damage by increasing apoptosis [140,141,142,143]. Obesity is also associated with a lower concentration of cardioprotective miR-221 in adipose tissue-derived EVs [104,136,144]. It can prevent cardiac IRI by attenuating apoptosis and reducing infarct size [145,146]. Furthermore, adipose tissue macrophage-derived EVs contain high miR-29a levels, which can promote insulin resistance in the peripheral tissues. This molecule mediates cardiac dysfunction in obesity-related cardiomyopathy and can be responsible for more extensive reperfusion injury. Its inhibition can attenuate heart damage and post-IRI fibrosis [127,147,148,149,150,151,152]. Another important exosomal miRNA in the development of obesity-related diabetes is miR-155, which increases cardiomyocyte apoptosis and the inflammatory response in the course of IRI. Inhibition of its delivery is reported as a promising therapy against both diabetes and cardiac injury [102,131,153,154,155]. A lower miR-26a level was noted in MVs from diabetic patients, which can contribute to more severe IRI due to the weaker inhibition of PTEN (phosphatase and tensin homolog deleted on chromosome ten). It decreases the pro-survival PI3K/AKT/mTOR (phosphoinositide 3-kinase/protein kinase B/mammalian target of rapamycin) signaling pathway activity and enhances apoptosis in cardiomyocytes in IRI [142,156,157]. Moreover, this molecule can reduce inflammatory cell infiltration and cytokine expression [158]. However, data showing the opposite indicates that knockdown of miR-26a can improve the cardiac function after reperfusion and can decrease cardiac apoptosis [159]. Wang et al. showed that hyperglycemia and hyperinsulinemia lead to the reduction of the cardioprotective miR-24 level [61,77]. This results in increased apoptosis, oxidative stress, a higher IRI area, and mortality [77]. Moreover, some exosomal miRNAs, which have been investigated as a diabetic biomarker (miR-23a, miR-192), can also play a role in apoptosis and oxidation stress regulation, and finally contributes to IRI [160,161,162,163]. 

Moreover, the therapy for hyperglycemia impacts the concentration of miRNA in EVs [164]. Insulin-induced hypoglycemia stimulates the secretion of endothelial cell-derived EVs (EVs peak in 240 min), which returns to baseline within 24 h [165]. Patients treated with metformin have miRNA profiles more similar to healthy controls than untreated patients [166]. Interestingly, metformin therapy is a well-documented form of protection against IRI in animal model. However, data from clinical trials are inconsistent [167,168,169,170]. Three months of therapy with another antidiabetic drug, acarbose, significantly decreased the number of platelet-derived MVs, which were initially elevated. This change can be associated with better outcomes after reperfusion [171,172]. 

Exosomal transport of all these molecules may be an important player in IRI in diabetic and obese patients and EV-targeted therapy is a promising method that may alleviate the negative effects of a dysregulated metabolism on the heart. Also, targeting the negative changes in adipose tissue during the obesity and diabetes may diminish IRI affecting the content of EVs originating from this tissue.

**Table 1:** A list of microRNAs which are increased in obesity and type 2 diabetes mellitus with their potential mechanisms of action and impact on ischemia-reperfusion injury.

Increased					
miRNA	Ob	DM2	Ref.	Effect	The Potential Regulatory Mechanism in IRI
miR-15b	Up	-	[136,173,174,175]	Aggravating	Increases apoptosis via downregulation of Bcl-2, MAPK3, and KCNJ2 [176,177,178]; inhibits the activity of the JAK2-STAT3 pathway, and promotes ROS production [179].
miR-23	Up	Up	[132,160]	Aggravating	Regulates glutamine metabolism, promotes the transformation of BMSCs into myocardial cells, suppresses expression of Manganese superoxide dismutase, enhances mitophagy, and inhibits connexin 43 expression [161,163,180,181].
miR-34	Up	Up	[91,136,173,182,183]	Aggravating	Increases apoptosis, infarct size, and suppresses angiogenesis silencing sirtuin-1 and Wnt/β-catenin signaling pathway, suppresses cardiomyocyte proliferation and cardiac recovery post-MI regulating cell cycle activity and death via modulation of its targets, including Bcl2, Cyclin D1, and SIRT1 [90,91,184,185,186,187,188].
miR-122	Up	Up	[132,136,173,189,190]	Aggravating	Promotes cardiomyocyte apoptosis via regulation of caspase 8 [191], inhibits the expression of Bcl-2, and upregulates the expression of HIF-1α, Bax, and caspase 9 via suppression of FOXP2 [192], downregulates the expression of the AKT/mTOR pathway, and upregulates the JNK/p38MAPK pathway [193].
miR-130	Up *	Up	[68,105,132,136,173,174,194,195,196]	Aggravating	Promotes worse cardiac function recovery, larger infarct size, and greater cardiomyocyte apoptosis targeting AMPKα1/α2, Birc6, and Ucp3 [68], increases NFκB-mediated inflammation and TGF-β1-mediated fibrosis via inhibition of PPAR-γ expression [197].
miR-155	Up	Up	[38,102,131,135,136,173]	Aggravating	Increases cardiomyocyte apoptosis in vitro via the downregulation of HIF-1α, RNA-binding protein Quaking, and SIRT1 [153,154,198], enhances the inflammatory response through the activation of the JAK2/STAT1 pathway [155], increases ROS generation during IRI [199].
miR-192	Up	Up	[132,136,160,173,200,201]	Aggravating	Induces apoptosis targeting FABP3, regulates oxidative stress in IRI [162,202]
miR-320	Up	Up	[37,132,135,200]	Aggravating	Increases infarct size and promotes apoptosis via the inhibition of AKIP1, IGF-1, HSP20, and AKT3 [137,138,139,203].
miR-483	Up	Up	[132,204,205]	Aggravating	Decreases cell viability and increases apoptosis by targeting the MDM4/p53 pathway [206], promotes apoptosis via the IGF-1 signaling pathway [207,208].
miR-21	Up *	Up *	[38,91,132,136,173,209,210]	Attenuating	Increases angiogenesis via silencing the cell death-inducing p53 target protein 1 [211], inhibits apoptosis via p38 downregulation and PI3k/Akt activation, decreases autophagy in the myocardial tissue via the AKT/mTOR pathway [91,152,212,213,214,215,216,217].
miR-26b	Up	Up	[132,136,174,196,218]	Attenuating	Reduces inflammation in IRI and improves myocardial remodeling via MAPK pathway activation [219], targets High Mobility Group AT-Hook 2 suppressing MI-induced fibrosis [220,221].
miR-142-3p	Up	Up	[105,136,195,200,201,222]	Attenuating	Inhibits IRI-induced cell apoptosis, autophagy, and fibrosis of the cardiomyocytes by targeting high mobility group box 1 and Rac Family Small GTPase 1 [223,224], improves cardiac function, and attenuates the myocardial inflammatory response targeting IRAK-1 [225].
miR-146	Up *	Up	[91,135,136,144,182,195,200,209,226,227]	Attenuating	Protects the myocardium from IRI by inhibition of NF-kB and TRAF6/p-p38/caspase-3 signaling pathways, targeting SMAD4, EGR1, and MED1; suppresses inflammatory cytokine production via IRAK-1 and TRAF6 [228,229,230,231,232], inhibits mitochondrial dysfunction in myocardial infarction by targeting cyclophilin D [233], regulates VEGF expression in the IRI heart [234].
miR-150	Up *	Up	[132,136,173,182,200]	Attenuating	Attenuates apoptosis and improves cardiac function via targeting Bax [235,236], reduces myocardial remodeling by downregulating Thioredoxin Interacting Protein [237].
miR-183	Up	-	[132]	Attenuating	Reduces infarct size and attenuates apoptosis through repressing voltage-dependent anion channel 1 expression, regulation of NF-κB signaling pathway, and suppression of p27 which activates the PI3K/AKT/FOXO3a signaling pathway [238,239,240].
miR-486	Up	Up	[136,174,241,242,243]	Attenuating	Inhibits apoptosis and improves cardiac function by suppressing PTEN expression, activating the PI3K/AKT signaling pathway [244,245], and targeting NDRG2 to inactivate the JNK/c-jun and NF-κB signaling pathways [246], promotes cardiac angiogenesis via fibroblastic MMP19-VEGFA cleavage signaling [247].
miR-29	Up	Up *	[132,136,173,200,201]	Ambiguous	Aggravating: Increasing apoptosis and fibrosis by suppression of Mcl-2 (Bcl-2 family), IGF-1, follistatin-like 1 protein, JAK2/STAT3 pathway, and the SIRT1/AMPK/PGC1α pathway [148,149,150,151,152,248];Attenuating: Inhibition of oxidative stress, apoptosis, and promotes the viability of cardiomyocytes with IRI in vivo model via downregulation of Cyclin T2 [249].
miR-143	Up	-	[136,189,195]	Ambiguous	Aggravating: Promotes cardiac ischemia-mediated mitochondrial impairment by the inhibition of protein kinase C epsilon [250], inhibits the mitosis of cardiomyocytes [251], promotes fibrosis via targeting sprouty3 [252];Attenuating: Promotes post-MI cell proliferation and reduced cell apoptosis in vitro via cyclooxygenase-2 [253]
miR-223	Up *	Up	[136,173,196,254,255,256]	Ambiguous	Aggravating: Increases cardiomyocyte apoptosis and oxidative stress by targeting KLF1 [257], enhances cardiac fibrosis after MI partially through targeting RASA1 [258], inhibits the angiogenesis of coronary microvascular endothelial cells in the ischemic heart [259]; Attenuating: Protects in vitro cells from hypoxia-induced apoptosis and excessive autophagy via the AKT/mTOR pathway by targeting PARP-1 [260], inhibits I/R-induced cardiac necroptosis at multiple layers [261].

(Ob—Obesity, DM2—diabetes mellitus type 2, *—indicates a contradictory finding where the miRNA was found to be down-regulated in at least one study). AKIP1—Aurora Kinase A Interacting Protein 1, AKT—protein kinase B, AMPKα1/α2—5’AMP-activated protein kinase α1/α2, Bax—Bcl-2-associated X protein, Bcl-2—B-cell lymphoma 2, Birc6—Baculoviral IAP Repeat Containing 6, EGR1—Early growth response protein 1, FABP—fatty acid binding protein, FOX—Forkhead Box Protein, HIF—hypoxia-inducible factor, HSP—heat shock protein, IGF—Insulin Growth Factor, IRAK1—Interleukin-1 receptor associated kinase, JAK—Janus Kinase, JNK—Jun N-terminal kinases, KCNJ2—Potassium Inwardly Rectifying Channel Subfamily J Member 2, KLF1—Kruppel Like Factor 1, MAPK3—Mitogen-Activated Protein Kinase 3, MDM4 – MDM4 Regulator of P53, MED1—Mediator Complex Subunit 1, MMP—matrix metalloproteinases, mTOR—mammalian target of rapamycin, NF-kB—nuclear factor kappa-light-chain-enhancer of activated B cells, NDGR2—NDRG Family Member 2, PGC-1α-Peroxisome proliferator-activated receptor gamma coactivator 1-alpha, PARP—Poly (ADP-ribose) polymerase, PI3K—Phosphoinositide 3-kinases, PPAR—peroxisome proliferator-activated receptor, RASA1—RAS P21 Protein Activator 1, ROS—reactive oxygen species, SIRT1—Sirtuin 1, SMAD4—SMAD Family Member 4, STAT—signal transducer and activator of transcription, TGF—Tumor Growth Factor, Ucp3—Uncoupling Protein 3, TRAF—TNF receptor associated factors, VEGF—Vascular endothelial growth factor.

**Table 2:** A list of microRNAs which are decreased in obesity and type 2 diabetes mellitus with their potential mechanisms of action and impact on ischemia-reperfusion injury.

Decreased					
miRNA	Ob	DM2	Ref.	Effect	The Potential Regulatory Mechanism in IRI
miR-17-5p	Down	Down	[136,189,200,201]	Aggravating	Promotes apoptosis induced by ER-stress and oxidative stress injury targeting TSG101 and STAT3 [262,263], increases apoptosis and vascular injury by suppressing the ERK pathway, and via downregulation of Bcl-2 in endothelium cells [264].
miR-24	-	Down	[77,200,265]	Attenuating	Inhibits apoptosis and excessive O-GlcNAcylation [61,77].
miR-126	Down	Down	[132,136,142,143,182,200,222,265]	Attenuating	Regulates oxidative stress and apoptosis via downregulation of ERRFI1 expression, and decline of PI3K/AKT pathway activity [140,141,266], decreases angiogenesis [152].
miR-132	Down	-	[136,189]	Attenuating	Inhibits apoptosis and ROS production via regulation of the TUG1/miR-132-3p/HDAC3 axis and through IL-1β downregulation [267,268,269], protects against oxygen and glucose deprivation via the inhibition of FOXO3a [270], enhances neovascularization [271].
miR-145	Down	Down	[136,189,272]	Attenuating	Inhibits IRI-induced apoptosis via regulation of the AKT3/mTOR and CaMKII-mediated ASK1 antiapoptotic signaling pathways, ameliorates inflammation by the NF-κB p65 pathway, and the negative regulation of CD40, protects the heart through induction of autophagy [273,274,275,276,277]; absence of miR-145 results in greater infarct thinning and dilatation [278].
miR-206	Down	-	[136,279]	Attenuating	Reduces IRI-induced apoptosis, targeting protein tyrosine phosphatase 1B, Gadd45β, and ATG3 (activating PI3K/Akt/mTOR pathway); reduces infarct size and improves cardiac function [236,280,281,282].
miR-221	Down	-	[104,105,136,144,194,241]	Attenuating	Reduces infarct size and prevents IRI-induced apoptosis via the PUMA/ETS-1 pathway and others [145,146].
miR-26a	-	Down	[142]	Ambiguous	Attenuating: Improves viability and inhibits apoptosis via regulation of the PTEN/PI3K/AKT signaling pathway, inhibition of high mobility group box 1 protein expression, inflammatory cell infiltration, and cytokine expression [156,158]. Aggravating: Possible proapoptotic mechanism via targeted regulation of the GSK3β/β-catenin signaling pathway [159]
miR-138	Down	-	[136,175,283]	Ambiguous	Attenuating: Reduces infarct size and myocardial I/R-induced mitochondrial apoptosis by targeting HIF-1α [284], increases the cardiac cells’ viability under hypoxia through targeting PDK1 [285]; Aggravating: Downregulation of miR-138-5p can regulate SIRT1 to inhibit cell pyroptosis and attenuate MI progression [286], miR-138 may mediate inhibition of hypoxia-induced proliferation of endothelial progenitor cells [287].

(Ob—Obesity, DM2—diabetes mellitus type 2). AKT—protein kinase B, ASK1—apoptosis signal-regulating kinase 1, Bcl-2—B-cell lymphoma 2, CaMKII—calcium/calmodulin-dependent protein kinase II, ER-endoplasmic reticulum, ERRFI1—ERBB Receptor Feedback Inhibitor 1, ERK—extracellular signal-regulated kinase, FOX—forkhead Box Protein, GSK3β—glycogen synthase kinase-3 beta, HDAC3—histone deacetylase 3, HIF—hypoxia-inducible factor, mTOR—mammalian target of rapamycin, NF-kB—nuclear factor kappa-light-chain-enhancer of activated B cells, PDK1—Pyruvate Dehydrogenase Kinase 1 PI3K—Phosphoinositide 3-kinases, PTEN—phosphatase and tensin homolog deleted on chromosome ten, SIRT1—Sirtuin 1, STAT—signal transducer and activator of transcription, TSG101—Tumor susceptibility gene 101 protein, TUG1—Taurine up-regulated 1 Gene.

### 3.2. Smoking

Myocardial infarction affects smokers ten years earlier than non-smokers [288]. Recently published research demonstrated an independent and strong association between smoking and intramyocardial hemorrhage occurrence in a group of STEMI patients [289]. The intramyocardial hemorrhage is a marker of severe ischemia-reperfusion injury caused by profound structural damage of the coronary microvasculature, which leads to extravasation of erythrocytes. Studies on cell culture showed that exposure to cigarette smoke affects the release and content of EVs [290,291,292,293,294,295,296,297]. It was noticed in a small group (*n* = 12) that active smoking of one cigarette can cause an immediate and significant increase in the number of MVs, which may be a sign of acute vascular injury [294]. Cordazzo et al. showed that cigarette smoke extract causes increased intracellular Ca^2+^ concentration, resulting in a higher generation of MVs [298]. In addition, CS-induced EVs may be enriched in MMP-14, proinflammatory cytokines (IL-1, IL-6, IL-8, MCP-1), procoagulant tissue factor, and phosphatidylserine which can cause destabilization of the atherosclerotic plaque, rapture, and occlusion of the artery as a consequence [299,300,301,302,303,304]. These vesicles can potentially enhance the IRI due to stimulation of inflammasome formation, which can contribute to pyroptosis and other types of cell death [305,306]. 

Cigarette smoke exposure may also impact the level of miRNA circulating inside EVs. For example, Fujita et al. noticed the upregulation of miR-210 which inhibits autophagy and exacerbates the IRI [307]. In another study, cigarette smoke-induced EVs were enriched in let-7d, miR-191, miR-126, and miR125a. The last two can alleviate the IRI by reducing apoptosis and the myocardial area after reperfusion, as mentioned above [308,309] (Table 1). The other two miRNA should be investigated as they may play a role in the IRI of other organs or may be important factors in cardiovascular diseases [310,311,312]. Further assessment of human serum has revealed that miR-29b is significantly increased and miR-223-3p is significantly decreased in smokers [313]. The role of these mi-RNAs is not clear. However, inhibition of miR-29b by dexmedetomidine was investigated and showed that it can reduce the IRI [314]. In addition, Zhang et al. noticed that LncRNA H19, which can inhibit miR-29b, decreases the reperfusion damage due to mediating the antiapoptotic effect of postconditioning [315]. Overexpression of miR-223 promotes oxidative stress, which can induce apoptosis by inhibiting the expression of FOXO3a [316]. On the other hand, this miRNA can have a cardioprotective effect. Some authors have shown that it could reduce IRI by suppressing necroptosis, apoptosis, and oxidative stress [257,260,261]. Other investigators, however, have not observed any significant difference in miRNA expression between smokers and non-smokers [317]. 

Studies show that also vaping is harmful to the endothelium, and it can cause a change in the concentration and content of EVs [318,319]. More studies are needed to assess the impact of substances released by electronic devices on IRI. 

### 3.3. Total Cholesterol Level

The number of EVs varies depending on the cholesterol level [122,320]. This correlation may be a result of obesity which is common among patients with hypercholesterolemia. Hypertensive-hypercholesterolemic hamsters have approximately 20 times the number of MVs than the control group with similar body mass, which suggests that the number of EVs can be independent of weight [321]. Nijiati et al. noticed that HDL-C levels and MVs are negatively correlated in humans after acute coronary syndrome [322]. In addition, incubation of platelets with native HDL3 reduces the number of MVs released [323]. Patients with familial hypercholesterolemia and increased oxidized LDL cholesterol levels have a higher number of pro-inflammatory MVs, which can promote fibrosis and necrosis after reperfusion [324]. 

One of the interventions used to reduce the cardiovascular risk associated with increased cholesterol level is statin therapy. The impact of this treatment was investigated in some studies. Two randomized trials have shown that perioperative therapy with statins can reduce myocardial IRI, but a large-scale study failed to confirm this. [325,326,327]. This effect may be mediated by EVs because, as some authors suggest, statin therapy impacts the level of EVs and consequently the IRI, but again this correlation has not been observed in all studies [328,329,330,331,332,333]. Interestingly, Kocsis et al. showed in an animal study that chronic and acute lovastatin therapy could have a different effect on conditioning. They noticed that chronic therapy decreased the preconditioning efficacy, whereas acute therapy decreased postconditioning efficacy [334]. A lower number of MVs, especially platelets, leukocytes, and endothelial cell-derived MVs have been demonstrated in patients treated with statins (simvastatin, rosuvastatin, atorvastatin) than in untreated patients [328]. This effect was not observed in diabetic patients on pitavastatin therapy [329]. The influence of statins on the shedding of MVs is greater with years of treatment [328]. Also, the effect of statins seems to be dose-dependent. It was shown that the number of MVs was significantly decreased in the 40-mg atorvastatin group compared with the 10-mg atorvastatin group (1683.75 ± 118.5 vs. 1847.98 ± 180.26 after one-year observation, baseline—2098.56 ± 223.73 vs. 2020.36 ± 206.94) [331]. As mentioned above, MVs may play an essential role in IRI, but this should be assessed by considering their origin and cargo, and not only their number. 

Simvastatin inhibits exosome production by reducing the levels of Alix, the protein that positively regulates the secretion of EVs [333,335]. In addition, this treatment led to a reduction in the levels of miR-150, which seems to be a key driver of endothelial migration (this process is an integral part of atherosclerotic plaque progression), which can make a patient more prone to MI [333,336]. And miR-150 may also be a regulator of cardiomyocyte survival during cardiac injury by directly repressing the proapoptotic gene EGR2 and reducing cell death after reperfusion [337].

Total cholesterol level and especially LDL level are essential cardiovascular risk factors that lead to death due to CV events. It is well known that statins have a cardioprotective effect, but their impact on IRI, especially with regard to EVs, still requires further studies.

### 3.4. Systolic Blood Pressure

Although arterial hypertension is one of the most common risk factors of higher mortality due to myocardial infarction, its impact on IRI is poorly studied [338]. Both hypertension and left ventricular hypertrophy induced by elevated arterial pressure are associated with increased myocardium vulnerability to IRI [339,340]. However, antecedent hypertension has no impact on reperfusion efficacy, infarct size, and reperfusion injury in magnetic resonance imaging, and is not significantly associated with one-year mortality [341,342]. Liu et al. characterized the differences in exosomal miRNA between hypertensive and normotensive rats using next-generation sequencing. They noticed that 27 types have significantly different expressions between the groups. Three of them—miR-17-5p, miR-15b-5p, and miR-486-5p—are strongly related to IRI’s myocardial susceptibility in animal studies. miR-17-5p and miR-15b-5p are overexpressed, whereas expression of miR-486-5p is decreased. miR-17-5p and miR-15b-5p aggravate IRI by activation of the MAPK/ERK pathway (mitogen-activated protein kinase/extracellular signal-regulated kinase) or by inhibition of antiapoptotic Potassium Inwardly Rectifying Channel Subfamily J Member 2 while miR-486-5p attenuates IRI by activation of the PI3K/Akt/mTOR pathway [178,244,245,262,264]. Therefore, circulating exosomal miRNA should be studied as a potential link between systolic blood pressure and increased susceptibility of the myocardium to IRI among hypertensive individuals.

### 3.5. Physical Effort

The lack of physical activity is a strong predictor of mortality, contributing to occurrence myocardial infarction. Physical exercise has many positive effects on cardiovascular health. Exercise training protects the myocardium against ischemia-reperfusion injury, reduces myocardial oxidative damage and improves cardiac function after IRI onset [343,344,345]. Various factors derived from the heart and other tissues are responsible for this effect, but much remains to be clarified. Physical activity increases the level of cardioprotective polypeptides in the heart, such as irisin, FGF21, and neuregulin, as well as non-coding RNA, such as miR-133. Physical activity also decreases the expression of miR-208, which can aggravate IRI [346]. The protective effect of exercise is also mediated by circulating transferable factors in plasma [347]. Recent studies have shown that EVs are one of those factors [346,348,349]. Exercise affects both the composition and the amount of EVs circulating in the serum [349,350,351,352]. The effect varies according to the type of activity as well as its duration and intensity [353]. Exercise-induced EVs attenuate myocardial ischemia-reperfusion injury and studies conducted so far indicate that these mechanisms are related to proteins and miRNAs. Bei et al. showed that the short-term beneficial effect of exercise on myocardial IRI is mainly caused by the increased number of circulating EVs and mediated by the activation of ERK1/2 and HSP27 signaling [59,348]. 

However, long-term protection provided by exercise-derived circulating EVs is also possible, and it is associated with differentially expressed miRNA. Long-term exercise training increases the level of exosomal miR-342-5p, which inhibits myocardial IRI-induced apoptosis targeting Caspase 9 and JNK2 and enhances survival signaling by PPM1f in cardiomyocytes [354]. Physical activity changes the expression of many other circulating and exosomal miRNAs [355,356,357,358,359], increasing some with a protective function against IRI, such as miR-17-3p, miR-25-3p, and miR-93-5p [360,361,362], or decreasing those aggravating IRI. The impact of exercise on many muscle-specific microRNAs with a significant role in IRI, such as miR-1, miR-21, miR-133, and miR-208, is still unclear and probably dependent on the type of activity [346,357,363,364]. Therefore, EVs are an important vehicle delivering exercise-dependent factors from distant and local tissues to cardiomyocytes. As endurance exercise-derived EVs have a therapeutic potential for metabolic diseases, they have become essential factors in the secondary prevention of myocardial infarction [365,366,367].

### 3.6. Sex

Males are more susceptible to cardiovascular diseases than females [368]. The impact of sex on the content of EVs has been rarely assessed. There are only a few analyses in which researchers evaluated these groups separately. EVs are more abundant in healthy women than men, but some studies have shown the opposite results [369,370,371]. The number of procoagulants microvesicles (phosphatidylserine and P-selectin positive) is higher in apparently healthy premenopausal women compared with age-matched men (32 ± 8.5 year-old women and 29 ± 3.2 year-old men), but this can be associated with anticonceptive therapy [369]. The growing body of data supports the idea that MVs can play a role in IRI [85,372,373]. The platelet-derived-MVs, which are also increased in age-matched females compared with males, can have a cardioprotective effect [372]. 

The cargo of EVs differs between the sexes. Bammert et al. showed that miR-125a expression was lower (∼215%; *p* < 0.05), and miR-34a was higher (∼210%; *p* < 0.05) in the EV of men than in women [374]. MiR-125a has a potential cardioprotective effect by ameliorating cardiomyocyte autophagy and oxidative injury. These mechanisms are activated by promoting gene expression dysregulation involved in cell death and apoptosis [308,309]. Furthermore, miR-34a, which is also dysregulated in DM and obesity, may play a role in IRI. The artificial inhibition of this miRNA can reduce the damage associated with reperfusion through the SIRT1 protective pathway [90]. This data supports the thesis that men are more susceptible to IRI than women. This can also be part of the explanation why the risk of death due to CV diseases is higher in men.

A notable increase has been observed in cardiovascular risk in women during midlife, a period coincident with menopause transition [375]. The differences between pre-menopausal and post-menopausal women are rarely investigated. The climacteric lowers the level of platelet-derived MVs but has no impact on the number of endothelial-derived MVs [376]. The effect of it on IRI is still not verified.

Interestingly, pregnancy also has an impact on the number and content of EVs. These differences play a role not only in embryo implantation and development [377], but can also regulate the function of the heart [378]. For example, EVs obtained from amniotic fluid mesenchymal stromal cells can reduce the IRI by stimulation of endothelial cell migration [379]. However, pregnant rats are more prone to myocardial IRI than non-pregnant rats due to a higher generation of ROS and activation of apoptosis [380]. The exact role of EVs among pregnant women in IRI is still under investigation.

### 3.7. Age

There is growing evidence that EVs can play a role in aging and age-related diseases [381]. Basic research has shown that age-related changes in the composition of EVs have an undisputed impact on IRI size.

For example, miR-128-3p in EVs (which inhibit the SMAD5 gene) was markedly upregulated in EVs obtained from older mice, and its inhibition by tongxinluo (a traditional Chinese medicine) reduced IRI in the cell culture of human cardiomyocytes [382,383]. The mechanism is still under investigation, but some data suggest that it can promote the Reperfusion Injury Salvage Kinase (RISK) pathway, which has a cardioprotective effect, by increasing serine/threonine kinase p70s6k1 expression [382,384]. 

The IL-1 signaling pathway plays an important role in IRI’s multiple mechanisms, including inflammation and apoptosis [385]. It was shown that older rats had a higher level of this protein in their EVs, which can be partly linked to age-related inflammatory conditions [386]. Administration of recombinant human IL-1 receptor antagonist significantly reduces cardiac fibrosis and increases myocardium viability in rats and mice [387,388,389]. It was recently demonstrated that a higher IL-1β level at admission in patients with acute MI was independently associated with the risk of mortality and recurrent MACEs (major adverse cardiovascular events), resulting from greater IRI susceptibility [390]. 

Fafián-Labora et el. observed a negative correlation between miR-146a, miR-132, and miR-155 levels in EVs and age. It was related to the downregulation of TLR4 in the targeted cells, which is a part of inflammaging [391]. This process can contribute to inflammation imbalance (a decline in the ability to respond to a new pathogen during infection with simultaneously increasing levels of proinflammatory cytokines such as IL-1), which can play a significant role in IRI due to increased apoptosis and fibrosis [392,393]. Unfortunately, there are only a few studies, mentioned above, which have investigated the direct relationship between inflammation and reperfusion injury.

Altered ROS removal appears to be the main reason for increased susceptibility to IRI [394]. EV-mediated delivery ofextracellular nicotinamide phosphoribosyltransferase decreases with aging and promotes the systemic decrease of the NAD+ level due to lower biosynthesis and higher consumption. As a consequence of these changes, ROS elimination is reduced, and oxidative stress is increased, which promotes IRI [395,396,397,398,399]. 

Only a few studies have evaluated the changes in the concentration and content of EVs with aging in humans. Recently, one research group noted a significant decline in EV concentration with age and increasing internalization by the cells. This change was also related to differences in content. They observed that p53, cleaved caspase-3, and PARP-1 decreased with age when some immune-related antigens such as MUC1, MUCIN-14, NY-ESO, CD-14, and PDL were increased. Apart from comparing these parameters between age groups, the authors checked them in individuals after five years and did not find significant differences in the concentration of 39 out of 46 assessed proteins in EVs. This means that the content is probably more a feature of an individual than the number is. However, the number of EVs significantly declined over this period [293,400]. Bæk et al. showed that the estrogen receptor level in EVs also decreased with aging for both genders, which is statistically significant only among men [293]. It would appear that these changes may be the recently discovered reason for the higher susceptibility of men and the elderly to IRI [12,401]. 

Finally, it was demonstrated that the mtDNA level encapsulated in EVs also decreases with age. Lazo et al. showed that the addition of EVs obtained from young donors to HeLa cells increases basal and maximal respiration levels. The rise was higher in comparison with cells incubated with EVs from older donors [402]. The data suggest that EVs support the mitochondrial function, and this regulation can change with aging thus influencing the IRI size [403]. The Galectin-3 (Gal-3) concentration was reduced in vesicles derived from the elderly [404]. Studies on animals showed that this protein has a cardioprotective function, and knock-out mice have increased apoptosis and decreased antioxidant defenses compared with WT (wild-type) mice at 24 h after reperfusion. The expression of proinflammatory proteins decreased in Gal-3 knockout mice compared with WT mice [405]. Longer observation (8 days) revealed that, compared with non-treated animals, the inhibition of Gal-3 by modified citrus peptide lowered myocardial inflammation and reduced fibrosis. The perfusion and compliance assessed by MRI was improved, and finally no differences in LV hypertrophy was observed [406]. This paradox is very interesting and suggests multiple roles of Gal-3 in IRI. 

The impact of aging on EVs is not in dispute, but the role of these changes on the cardiovascular system should draw the attention of researchers. Further studies may help to find interventions, associated with EVs, that can decrease the risk of cardiovascular death among older people.

## 4. Conclusions

**Figure 3:**
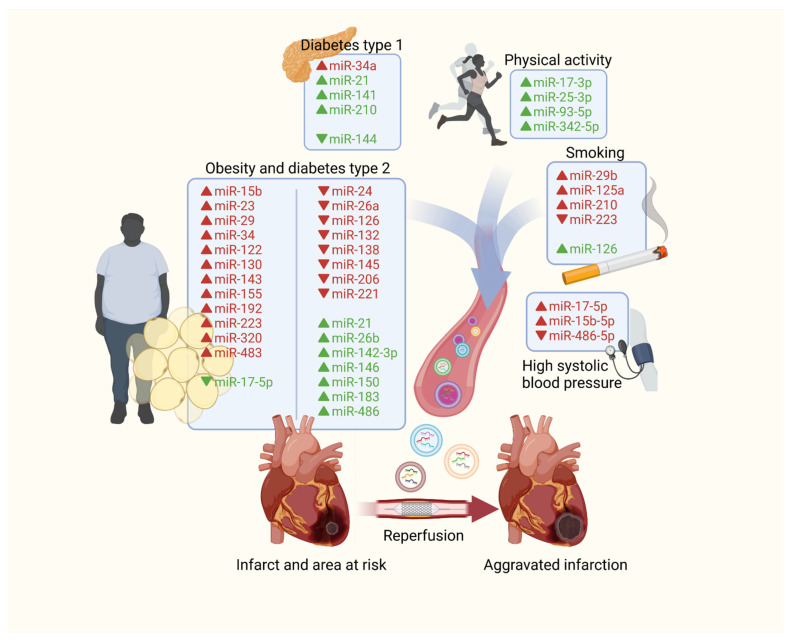
Impact of the main cardiovascular risk factors and physical activity on EVs’ expression of miRNAs and its potential influence on myocardial susceptibility to IRI. (upward arrow—increased level, downward arrow—decreased level, green—attenuating IRI, red—aggravating IRI).

The results of both experimental and clinical studies indicate that modifiable as well as non-modifiable cardiovascular risk factors change the number and content of EVs of various origin. These differences may have a negative impact on intercellular signalling, thus influencing the vulnerability of the heart to IRI. There are many potential components of EVs that may participate in this complicated crosstalk between the peripheral tissue and the heart, however, miRNAs seem to be the most important (Figure 3). Also, some cellular origins of EVs like adipose tissue appear to be more significant than the others.

Based on this information, it can be concluded that non-pharmacological interventions such as physical activity, body weight reduction, smoke cessation, and better control of systolic blood pressure may have beneficial effects on the myocardium and its susceptibility to IRI by modification of the composition and quantity of EVs. Moreover, EVs may be a new target for long-term management in secondary prophylaxis of acute coronary syndrome. Perhaps, the detection of circulating EVs for the prediction and prognosis of patient outcome after MI onset or strategies of blocking abnormal production of EVs could have great clinical implications in the future. However, randomized-controlled trials are needed to verify this hypothesis.

## Data Availability

Not applicable.
